# *N*,*N*′-Bis[(*E*)-1-(thio­phen-3-yl)ethyl­idene]ethane-1,2-diamine

**DOI:** 10.1107/S1600536812009798

**Published:** 2012-03-10

**Authors:** Abdullah M. Asiri, Hassan M. Faidallah, Khalid A. Khan, Seik Weng Ng, Edward R. T. Tiekink

**Affiliations:** a Chemistry Department, Faculty of Science, King Abdulaziz University, PO Box 80203, Jeddah, Saudi Arabia; b The Center of Excellence for Advanced Materials Research, King Abdulaziz University, Jeddah, PO Box 80203, Saudi Arabia; c Department of Chemistry, University of Malaya, 50603 Kuala Lumpur, Malaysia

## Abstract

The complete mol­ecule of the title compound, C_14_H_16_N_2_S_2_, is generated by a crystallographic inversion centre. The thio­phene residue is close to being coplanar with the imine group [C—C—C—N torsion angle = 6.5 (2)°], and the conformation about the imine C=N bond [1.281 (2) Å] is *E*. In the crystal, the three-dimensional architecture is consolidated by C—H⋯N, C—H⋯π and S⋯S [3.3932 (7) Å] inter­actions.

## Related literature

For background to 2-substituted thio­phenes, see: Kleemann *et al.* (2006 ▶). For related structures, see: Prasath *et al.* (2010*a* ▶,*b* ▶).
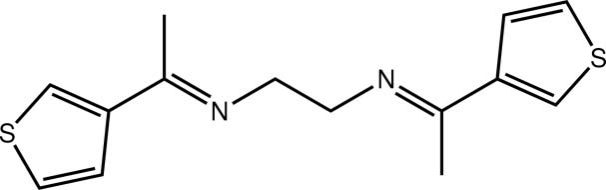


## Experimental

### 

#### Crystal data

C_14_H_16_N_2_S_2_
*M* *_r_* = 276.41
Monoclinic, 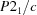

*a* = 7.5231 (6) Å
*b* = 11.2338 (6) Å
*c* = 8.5967 (6) Å
β = 112.894 (9)°
*V* = 669.30 (8) Å^3^
*Z* = 2
Mo *K*α radiation
μ = 0.38 mm^−1^
*T* = 100 K
0.20 × 0.15 × 0.10 mm

#### Data collection

Agilent SuperNova Dual diffractometer with an Atlas detector
Absorption correction: multi-scan (*CrysAlis PRO*; Agilent, 2011 ▶) *T*_min_ = 0.928, *T*_max_ = 0.963
2789 measured reflections
1542 independent reflections
1339 reflections with *I* > 2σ(*I*)
*R*_int_ = 0.033

#### Refinement

*R*[*F* ^2^ > 2σ(*F* ^2^)] = 0.039
*wR*(*F* ^2^) = 0.105
*S* = 1.06
1542 reflections
83 parameters
H-atom parameters constrained
Δρ_max_ = 0.45 e Å^−3^
Δρ_min_ = −0.41 e Å^−3^

### 

Data collection: *CrysAlis PRO* (Agilent, 2011 ▶); cell refinement: *CrysAlis PRO*; data reduction: *CrysAlis PRO*; program(s) used to solve structure: *SHELXS97* (Sheldrick, 2008 ▶); program(s) used to refine structure: *SHELXL97* (Sheldrick, 2008 ▶); molecular graphics: *ORTEP-3* (Farrugia, 1997 ▶) and *DIAMOND* (Brandenburg, 2006 ▶); software used to prepare material for publication: *publCIF* (Westrip, 2010 ▶).

## Supplementary Material

Crystal structure: contains datablock(s) global, I. DOI: 10.1107/S1600536812009798/hb6669sup1.cif

Structure factors: contains datablock(s) I. DOI: 10.1107/S1600536812009798/hb6669Isup2.hkl

Supplementary material file. DOI: 10.1107/S1600536812009798/hb6669Isup3.cml

Additional supplementary materials:  crystallographic information; 3D view; checkCIF report

## Figures and Tables

**Table 1:** Hydrogen-bond geometry (Å, °) *Cg*1 is the centroid of the S1,C1–C4 ring.

*D*—H⋯*A*	*D*—H	H⋯*A*	*D*⋯*A*	*D*—H⋯*A*
C1—H1⋯N1^i^	0.95	2.51	3.454 (2)	172
C6—H6*C*⋯*Cg*1^ii^	0.98	2.74	3.624 (2)	150

Symmetry codes: (i) 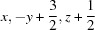; (ii) 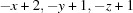.

**Fig. 1.**
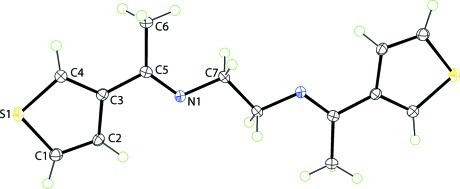
The molecular structure of (I) showing displacement ellipsoids at the 50% probability level. The unlabelled atoms are related by the symmetry operation (1-x, 1-y, -z).

**Fig. 2.**
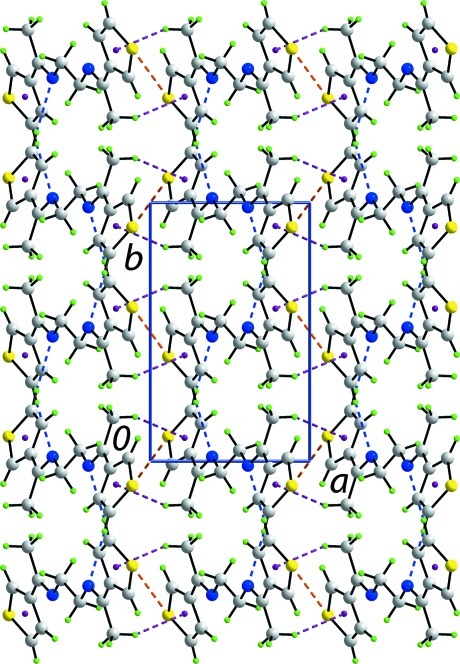
A view in projection down the c axis of the unit-cell contents of (I). The C—N···N, C—H···π and S···S interactions are shown as blue, purple and orange dashed lines, respectively.

## supplementary crystallographic information

### Comment

Thiophenes attract attention for their biological activity amongst other properties (Kleemann *et al.*, 2006). In continuation of structural studies of thienyl derivatives (Prasath *et al.*, 2010*a*; Prasath *et al.*, 2010*b*), herein the title compound, bis[1-(thiophen-3-yl)ethylidene]ethane-1,2-diamine (I), is described.

The asymmetric unit in (I), Fig. 1, comprises half a molecule with the full molecule generated by a crystallographic centre of inversion. The thiophene residue is co-planar with the imine group as seen in the value of the C2—C3—C5—N1 torsion angle of 6.5 (2) °. In fact the entire molecule is planar with the r.m.s. deviation for the 18 non-hydrogen atoms being 0.068 Å; the maximum deviations are found for the S1 [0.092 (1) Å] and C2 [-0.099 (2) Å] atoms. The conformation about the imine N1—C5 bond [1.281 (2) Å] is *E*.

In the crystal packing the molecules associate *via* C—H···N, C—H···π, [Table 1] and S···S [S1···S1^i^ = 3.3932 (7) Å for *i*: 2 - *x*, 1 - *y*, 2 - *z*] interactions to form a three-dimensional architecture, Fig. 2.

### Experimental

A mixture of ethylenediamine (0.6 g, 0.01 *M*) and 2-acetyl thiophene (0.7 g, 0.01 *M*) in dry benzene (50 ml) was refluxed using a Dean-Stark trap until no more water was collected (2 h). The benzene was then removed under reduced pressure and the residue treated with methanol. The solid that separated out was recrystallized from ethanol as colourless prisms. Yield: 72%. *M*.pt: 405–407 K.

### Refinement

Carbon-bound H-atoms were placed in calculated positions [C—H = 0.95 to 0.99 Å, *U*_iso_(H) = 1.2 to 1.5*U*_eq_(C)] and were included in the refinement in the riding model approximation.

### Crystal data

**Table:**

C_14_H_16_N_2_S_2_	*F*(000) = 292
*M**_r_* = 276.41	*D*_x_ = 1.372 Mg m^−^^3^
Monoclinic, *P*2_1_/*c*	Mo *K*α radiation, λ = 0.71073 Å
Hall symbol: -P 2ybc	Cell parameters from 1637 reflections
*a* = 7.5231 (6) Å	θ = 2.6–27.5°
*b* = 11.2338 (6) Å	µ = 0.38 mm^−^^1^
*c* = 8.5967 (6) Å	*T* = 100 K
β = 112.894 (9)°	Prism, colourless
*V* = 669.30 (8) Å^3^	0.20 × 0.15 × 0.10 mm
*Z* = 2	

### Data collection

**Table:**

Agilent SuperNova Dual diffractometer with an Atlas detector	1542 independent reflections
Radiation source: SuperNova (Mo) X-ray Source	1339 reflections with *I* > 2σ(*I*)
Mirror monochromator	*R*_int_ = 0.033
Detector resolution: 10.4041 pixels mm^-1^	θ_max_ = 27.6°, θ_min_ = 2.9°
ω scan	*h* = −9→9
Absorption correction: multi-scan (*CrysAlis PRO*; Agilent, 2011)	*k* = −14→9
*T*_min_ = 0.928, *T*_max_ = 0.963	*l* = −11→7
2789 measured reflections	

### Refinement

**Table:**

Refinement on *F*^2^	Primary atom site location: structure-invariant direct methods
Least-squares matrix: full	Secondary atom site location: difference Fourier map
*R*[*F*^2^ > 2σ(*F*^2^)] = 0.039	Hydrogen site location: inferred from neighbouring sites
*wR*(*F*^2^) = 0.105	H-atom parameters constrained
*S* = 1.06	*w* = 1/[σ^2^(*F*_o_^2^) + (0.0482*P*)^2^ + 0.3751*P*] where *P* = (*F*_o_^2^ + 2*F*_c_^2^)/3
1542 reflections	(Δ/σ)_max_ = 0.001
83 parameters	Δρ_max_ = 0.45 e Å^−^^3^
0 restraints	Δρ_min_ = −0.41 e Å^−^^3^

### Special details

**Table:**

Geometry. All e.s.d.'s (except the e.s.d. in the dihedral angle between two l.s. planes) are estimated using the full covariance matrix. The cell e.s.d.'s are taken into account individually in the estimation of e.s.d.'s in distances, angles and torsion angles; correlations between e.s.d.'s in cell parameters are only used when they are defined by crystal symmetry. An approximate (isotropic) treatment of cell e.s.d.'s is used for estimating e.s.d.'s involving l.s. planes.
Refinement. Refinement of *F*^2^ against ALL reflections. The weighted *R*-factor *wR* and goodness of fit *S* are based on *F*^2^, conventional *R*-factors *R* are based on *F*, with *F* set to zero for negative *F*^2^. The threshold expression of *F*^2^ > σ(*F*^2^) is used only for calculating *R*-factors(gt) *etc*. and is not relevant to the choice of reflections for refinement. *R*-factors based on *F*^2^ are statistically about twice as large as those based on *F*, and *R*- factors based on ALL data will be even larger.

### Fractional atomic coordinates and isotropic or equivalent isotropic displacement parameters (Å^2^)

**Table:**

	*x*	*y*	*z*	*U*_iso_*/*U*_eq_	
S1	0.88734 (7)	0.59544 (4)	0.83858 (6)	0.01735 (17)	
N1	0.6122 (2)	0.51740 (13)	0.23310 (18)	0.0129 (3)	
C1	0.7443 (3)	0.69397 (16)	0.6897 (2)	0.0161 (4)	
H1	0.7138	0.7719	0.7143	0.019*	
C2	0.6805 (3)	0.64614 (16)	0.5325 (2)	0.0138 (4)	
H2	0.5985	0.6872	0.4342	0.017*	
C3	0.7493 (2)	0.52741 (15)	0.5294 (2)	0.0127 (4)	
C4	0.8632 (3)	0.48878 (16)	0.6889 (2)	0.0151 (4)	
H4	0.9214	0.4123	0.7133	0.018*	
C5	0.7023 (2)	0.45986 (15)	0.3700 (2)	0.0121 (4)	
C6	0.7668 (3)	0.33137 (16)	0.3829 (2)	0.0156 (4)	
H6A	0.6939	0.2903	0.2764	0.023*	
H6B	0.7435	0.2923	0.4751	0.023*	
H6C	0.9049	0.3283	0.4055	0.023*	
C7	0.5591 (3)	0.45805 (16)	0.0702 (2)	0.0144 (4)	
H7A	0.4831	0.3856	0.0675	0.017*	
H7B	0.6771	0.4338	0.0537	0.017*	

### Atomic displacement parameters (Å^2^)

**Table:**

	*U*^11^	*U*^22^	*U*^33^	*U*^12^	*U*^13^	*U*^23^
S1	0.0200 (3)	0.0185 (3)	0.0112 (3)	0.00100 (18)	0.00349 (19)	−0.00088 (17)
N1	0.0144 (8)	0.0129 (7)	0.0110 (7)	−0.0001 (6)	0.0043 (6)	−0.0005 (6)
C1	0.0183 (9)	0.0150 (8)	0.0170 (9)	0.0011 (7)	0.0091 (7)	0.0012 (7)
C2	0.0147 (9)	0.0151 (9)	0.0128 (8)	0.0013 (7)	0.0065 (7)	0.0023 (7)
C3	0.0124 (8)	0.0129 (8)	0.0134 (9)	−0.0012 (7)	0.0057 (7)	0.0010 (7)
C4	0.0171 (9)	0.0137 (8)	0.0136 (9)	0.0003 (7)	0.0052 (7)	0.0001 (7)
C5	0.0101 (8)	0.0123 (8)	0.0146 (9)	−0.0011 (6)	0.0055 (7)	−0.0006 (7)
C6	0.0184 (9)	0.0125 (8)	0.0158 (9)	0.0020 (7)	0.0064 (7)	0.0004 (7)
C7	0.0177 (9)	0.0124 (8)	0.0121 (9)	0.0005 (7)	0.0047 (7)	−0.0024 (7)

### Geometric parameters (Å, º)

**Table:**

S1—C4	1.7154 (18)	C3—C5	1.484 (2)
S1—C1	1.7174 (19)	C4—H4	0.9500
N1—C5	1.281 (2)	C5—C6	1.513 (2)
N1—C7	1.460 (2)	C6—H6A	0.9800
C1—C2	1.357 (2)	C6—H6B	0.9800
C1—H1	0.9500	C6—H6C	0.9800
C2—C3	1.435 (2)	C7—C7^i^	1.517 (3)
C2—H2	0.9500	C7—H7A	0.9900
C3—C4	1.374 (2)	C7—H7B	0.9900
			
C4—S1—C1	92.19 (9)	N1—C5—C6	126.06 (15)
C5—N1—C7	120.01 (15)	C3—C5—C6	117.77 (15)
C2—C1—S1	111.31 (14)	C5—C6—H6A	109.5
C2—C1—H1	124.3	C5—C6—H6B	109.5
S1—C1—H1	124.3	H6A—C6—H6B	109.5
C1—C2—C3	113.35 (16)	C5—C6—H6C	109.5
C1—C2—H2	123.3	H6A—C6—H6C	109.5
C3—C2—H2	123.3	H6B—C6—H6C	109.5
C4—C3—C2	111.37 (16)	N1—C7—C7^i^	109.65 (18)
C4—C3—C5	126.30 (16)	N1—C7—H7A	109.7
C2—C3—C5	122.32 (15)	C7^i^—C7—H7A	109.7
C3—C4—S1	111.78 (14)	N1—C7—H7B	109.7
C3—C4—H4	124.1	C7^i^—C7—H7B	109.7
S1—C4—H4	124.1	H7A—C7—H7B	108.2
N1—C5—C3	116.16 (15)		
			
C4—S1—C1—C2	0.34 (14)	C7—N1—C5—C3	−179.72 (15)
S1—C1—C2—C3	−0.7 (2)	C7—N1—C5—C6	1.3 (3)
C1—C2—C3—C4	0.8 (2)	C4—C3—C5—N1	−172.11 (17)
C1—C2—C3—C5	−177.97 (16)	C2—C3—C5—N1	6.5 (2)
C2—C3—C4—S1	−0.6 (2)	C4—C3—C5—C6	7.0 (3)
C5—C3—C4—S1	178.19 (14)	C2—C3—C5—C6	−174.39 (15)
C1—S1—C4—C3	0.13 (15)	C5—N1—C7—C7^i^	175.58 (18)

Symmetry code: (i) −*x*+1, −*y*+1, −*z*.

### Hydrogen-bond geometry (Å, º)

Cg1 is the centroid of the S1,C1–C4 ring.

**Table:**

*D*—H···*A*	*D*—H	H···*A*	*D*···*A*	*D*—H···*A*
C1—H1···N1^ii^	0.95	2.51	3.454 (2)	172
C6—H6*C*···*Cg*1^iii^	0.98	2.74	3.624 (2)	150

Symmetry codes: (ii) *x*, −*y*+3/2, *z*+1/2; (iii) −*x*+2, −*y*+1, −*z*+1.
